# A comparative study of explainability methods for whole slide classification of lymph node metastases using vision transformers

**DOI:** 10.1371/journal.pdig.0000792

**Published:** 2025-04-15

**Authors:** Jens Rahnfeld, Mehdi Naouar, Gabriel Kalweit, Joschka Boedecker, Estelle Dubruc, Maria Kalweit

**Affiliations:** 1 Collaborative Research Institute Intelligent Oncology (CRIION), Freiburg, Germany; 2 University of Freiburg, Freiburg, Germany; 3 BrainLinks-BrainTools, Freiburg, Germany; 4 Department of Pathology, University Hospital and University of Lausanne, Lausanne, Switzerland; University of Illinois Urbana-Champaign, UNITED STATES OF AMERICA

## Abstract

Recent advancements in deep learning have shown promise in enhancing the performance of medical image analysis. In pathology, automated whole slide imaging has transformed clinical workflows by streamlining routine tasks and diagnostic and prognostic support. However, the lack of transparency of deep learning models, often described as *black boxes*, poses a significant barrier to their clinical adoption. This study evaluates various explainability methods for Vision Transformers, assessing their effectiveness in explaining the rationale behind their classification predictions on histopathological images. Using a Vision Transformer trained on the publicly available CAMELYON16 dataset comprising of 399 whole slide images of lymph node metastases of patients with breast cancer, we conducted a comparative analysis of a diverse range of state-of-the-art techniques for generating explanations through heatmaps, including Attention Rollout, Integrated Gradients, RISE, and ViT-Shapley. Our findings reveal that Attention Rollout and Integrated Gradients are prone to artifacts, while RISE and particularly ViT-Shapley generate more reliable and interpretable heatmaps. ViT-Shapley also demonstrated faster runtime and superior performance in insertion and deletion metrics. These results suggest that integrating ViT-Shapley-based heatmaps into pathology reports could enhance trust and scalability in clinical workflows, facilitating the adoption of explainable artificial intelligence in pathology.

## Author summary

The objective of our research was to investigate methods for enhancing the explainability of state-of-the-art Vision Transformer models for medical image analysis in histopathology. These models are often perceived as opaque, lacking transparency, which can limit their deployment in clinical settings. In our study, we evaluated various approaches for generating visual explanations that highlight critical areas in histopathology images that influence the Vision Transformers’ decisions and serve as heatmaps for pathologists. By comparing different techniques, we identified that the ViT-Shapley method generated the most reliable and effective heatmaps. In evaluating the practical impact, we considered both clinical usability and computational efficiency. These heatmaps can help clinicians understand and trust the model’s decisions, potentially integrating these advanced AI tools into routine clinical workflows to improve diagnostic support. The findings of our study indicate that the integration of Transformer-based models with effective explainability methods can enhance the accuracy and transparency of automated whole slide image analysis, ultimately benefiting patient care.

## Introduction

Recent advances in deep learning (DL) [1–3] have enhanced medical image analysis, including the classification of gigapixel Whole Slide Images (WSIs) in histopathology [4–6]. The impact of automated WSI examination is immense in terms of resources, quality and clinical decision support, leading to a strong current to integrate these technologies into clinical workflows. The ability of deep neural networks to process and analyze large amounts of data can assist human pathologists in inspecting WSIs to enhance clinical workflows and accuracy, potentially detecting patterns within WSIs that may not be immediately apparent to the human eye and facilitating a more comprehensive assessment of the slides [7,8]. Transformers [9], which use self-attention mechanisms to assign importance to different parts of the input, have set new benchmarks in natural language processing. In the vision domain, Vision Transformers (ViT) are increasingly being applied to image analysis [10] and medical image classification [11], showing improvements in the qualitative accuracy of the classification of WSIs [2]. Unlike classical models, which struggle with high-dimensional inputs like images due to their large number of features, (Vision) Transformers can effectively handle and learn from such data, achieving very high prediction accuracy.

However, in the field of medicine, practitioners require not only accurate predictions but also an understanding of the reasoning behind these predictions to ensure trust and the effective use of such technology. While classical machine learning (ML) approaches, such as Logistic Regression models or Decision Trees, are often easier to understand and inherently more explainable due to their simpler structures and explicit decision paths, deep learning models, especially Transformers, present significant challenges in terms of explainability. The complexity and lack of transparency inherent in Transformer models make it difficult for humans to discern how these models derive their conclusions and pose substantial obstacles for clinical acceptance [12].

Explainable AI (xAI) methods [13] have been developed to enhance the transparency of AI models, particularly Transformer-based deep neural networks. Among these methods, *attribution methods* play a crucial role. Attribution methods attempt to assign an importance score to each input feature (e.g., pixels or regions in an image), indicating how much each feature contributes to the model’s prediction. To compute such scores, several techniques can be employed. These include analyzing gradients to see how changes in input affect outputs, examining model attention to highlight relevant features or the use of removal-based methods which probe how the model prediction changes under masked or altered images. The outputs of attribution methods are often visualized as *attribution maps* or *heatmaps*, where each region of the input image is colored according to its attributed importance. In these heatmaps, areas of the input image are colored in shades of red and blue. Red areas indicate that those parts of the image are important for the model’s predictions, while blue areas suggest less importance. This color coding helps medical experts quickly identify which features of the image are most significant in the decision-making process.

However, despite the availability of a range of explainability methods, their application to Transformer models in the context of WSI classification in oncology remains limited. Existing studies have not fully addressed how these methods scale and perform when applied to the gigapixel images typical in histopathology, nor have they thoroughly evaluated the resource requirements associated with their implementation. To address this gap, our study focuses on the comparative analysis of state-of-the-art explainability methods applied to Transformers for the classification of WSIs in lymph node metastases among women with breast cancer.

We benchmarked current attribution methods including Attention Rollout with residuals [14], which aggregates attention weights across layers to generate attention maps; Integrated Gradients [15], a gradient-based method that attributes predictions by integrating gradients along the path from a baseline to the input; and RISE [16], which creates importance maps by applying random masks to the input images and observing changes in the output. Additionally, we evaluated ViT-Shapley [17], which utilizes approximate Shapley values to attribute model predictions in Vision Transformers, extending the experimental framework proposed in [18]. For evaluation, we used the publicly available *CAMELYON16* dataset [19] consisting of WSIs of lymph node sections from patients with breast cancer. This dataset is a standardized benchmark, which includes a comprehensive representation of the histopathological variability present in breast cancer metastases and provides a robust foundation for evaluating the performance of explainability methods in a clinically relevant context. Using the trained classifier developed by Naouar et al. [20], we generated attribution maps for gigapixel WSIs resulting from the methods described above. Through comprehensive qualitative and quantitative assessments, we compared the performance and resource requirements of these methods. Our key contributions are as follows:

1. We demonstrate the feasibility of generating meaningful attribution maps for gigapixel WSIs using Vision Transformers and the aforementioned attribution methods.
2. We provide a thorough comparison of the scalability and resource requirements of RISE and ViT-Shapley.
3. We identify the ViT-Shapley method as the most effective approach among those evaluated, outperforming others in generating concise and clinically relevant attribution maps while also being computationally efficient.

By enhancing the explainability and trustworthiness of expressive Transformer-based black-box classifiers for sentinel lymph node sections from patients with breast cancer, our study bridges the gap between advanced AI models and clinical practice. To the best of our knowledge, this is the first comprehensive study to evaluate and compare the scalability of explainability methods for Transformers in this area.

## Results

The study utilized the publicly available CAMELYON16 dataset, consisting of 399 hematoxylin and eosin (H&E) stained WSIs of sections of sentinel lymph nodes. More details can be found in the *Dataset Description* and *Dataset Availability* (sub-)sections. As classifier model, we used a Vision Transformer model trained on 20 ×  magnification [20], which had an AUROC (Area Under the Receiver Operating Characteristic Curve; a measure of the model’s ability to differentiate between classes, where 100% indicates perfect distinction) of 96 . 3 ± 0 . 1 and an MCC (Matthews Correlation Coefficient; an overall measure of prediction quality ranging from -100% to 100% that accounts for true and false positives and negatives, where 100% is perfect prediction even with imbalanced data) of 55 . 16  ±  1 . 11. Additionally, it had a precision of 48 . 02 ± 1 . 73, a recall of 80 . 23 ± 0 . 81 and a specificity of 98 . 72 ± 0 . 14. On this basis, we assessed the capabilities of several explainability methods for this classifier: Attention Rollout with and without residuals, Integrated Gradients, RISE with both binominal and uniform mask sampling, and ViT-Shapley. In the *Materials and Methods* section, we provide an in-depth explanation of how these different methods work.

### Qualitative assessment

**Fig 1:**
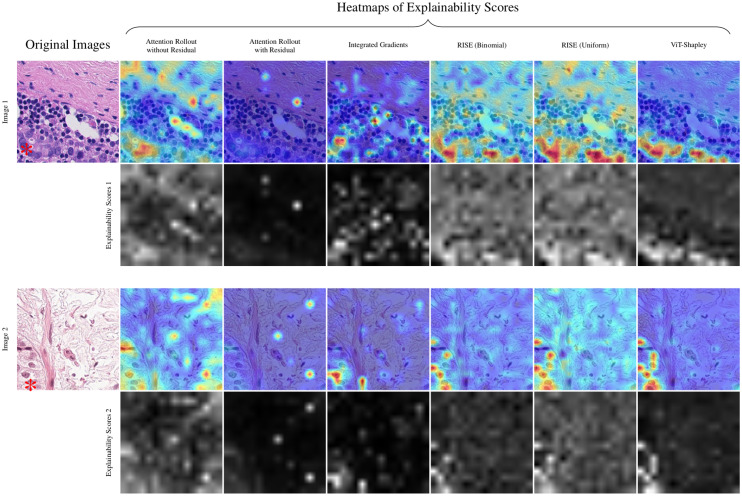
Qualitative examples for different heatmaps of explainability scores from WSIs test_110 and test_052. Original images are shown on the left, with metastatic cells on the lower part. Tumor tissue is marked with *. The first row shows each method’s explainability scores as heatmaps blended over the image, and the second row the corresponding raw explainability scores inferred from the trained Vision Transformer. ViT-Shapley (on the right) shows the clearest distinction between tumoral and non-tumoral tissue. Original images can be retrieved from https://camelyon17.grand-challenge.org/Data/.

**Fig 2:**
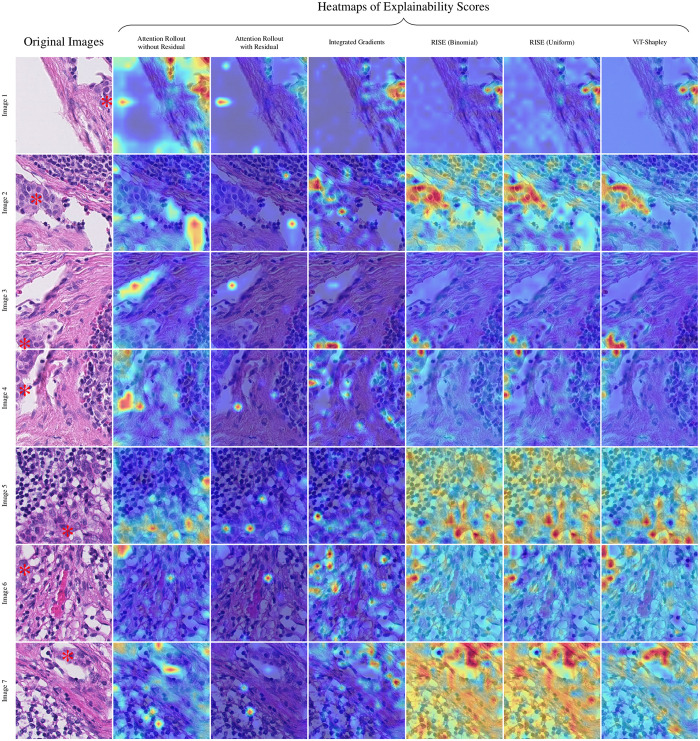
Further qualitative examples for patches from WSIs test_004 and test_008. Tumor tissue is marked with * in the original image. Each method’s explainability scores are shown as heatmaps blended over the image. Original images can be retrieved from https://camelyon17.grand-challenge.org/Data/.

**Fig 3:**
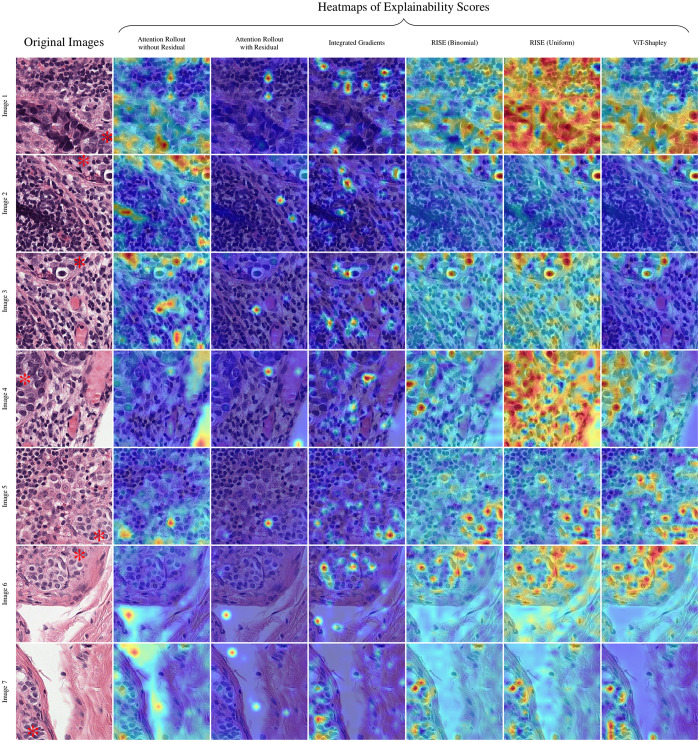
Further qualitative examples for patches from WSIs test_010 and test_013. For every image, each method’s explainability scores as heatmaps are blended over the image. Tumor tissue is marked with *. Original images can be retrieved from https://camelyon17.grand-challenge.org/Data/.

The qualitative assessment was conducted by an expert pathologist. The aim was to assess the logical coherence of the heatmaps identified by the examined explainability methods, which ought to highlight regions determined to be important for the decision of the trained Vision Transformer classifiers. Visual analysis showed (cf. Figs 1 to 3), that – despite highlighting some of the tumor cells – Attention Rollout methods tend to highlight overconfident artifacts, emphasizing non-informative regions, such as the background. The incorporation of residuals intensified these effects. Integrated Gradients primarily focused on a subset of individual tumor cells. In contrast, both RISE and ViT-Shapley generally highlighted areas extensively covered by tumor cells. While RISE distributes more importance and variance to background regions, ViT-Shapley shows the most concised heatmaps with strongest focus on the complete set of visible tumor cells.

### Quantitative evaluation

For a quantitative evaluation, we focus on the widely used *Insertion* and *Deletion* metrics [16], for which we generate predictions while inserting or removing features in the order of most to least importance and evaluate the area under the curve of prediction probabilities. Due to computational limitations and the noisy nature of the Faithfulness metric [21], we omit this metric for the *CAMELYON16* dataset.

**Fig 4:**
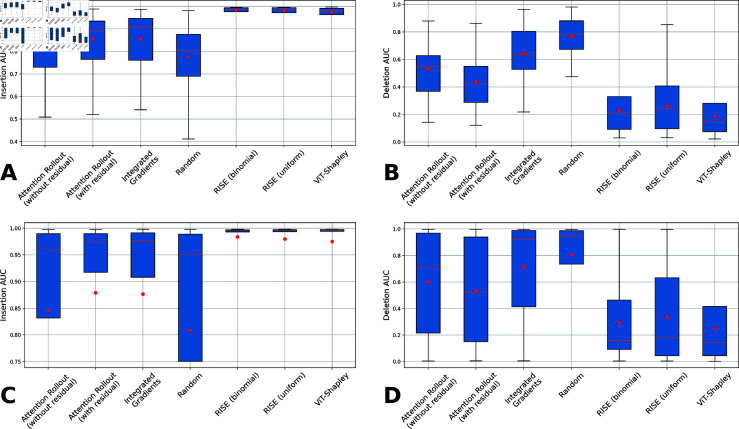
Mean Insertion and Deletion scores on the basis of WSIs and patches. (A) Mean over per WSI averaged AUC. (B) Deletion (*↓*): Mean over per WSI averaged AUC. (C) Insertion (*↑*): Mean over all individual patch AUCs. (D) Deletion (*↓*): Mean over all individual patch AUCs. Red dot is the mean within the populations.

Our analysis reveals consistent patterns across methods, as depicted in Fig 4 and detailed in Table 1. Initial analyses confirmed that all evaluated methods outperformed random attribution maps significantly (${}^{****}p<1.65$  ⋅  $10^{-36}$; Welch’s t-test). Attention Rollout, enhanced with residuals in the attention matrix, exhibited superior performance in both metrics. Integrated Gradients achieved comparable Insertion scores to Attention Rollout but fell short in Deletion metrics (${}^{*}p<0.0465$; Welch’s t-test). Notably, removal-based methods demonstrated significantly superior performance (${}^{***}p<4.61$  ⋅  10^−4^; Welch’s t-test). RISE, with binomially and uniformly distributed mask cardinalities, showed marginally higher AUC for Insertion (${}^{****}p<1.33$  ⋅  $10^{-7}$; Welch’s t-test), while ViT-Shapley’s lower Deletion AUC indicated better performance compared to both RISE variants (${}^{****}p<7.13$  ⋅  $10^{-47}$; Welch’s t-test). Remarkably, RISE performed better with binomial distribution of mask cardinality (${}^{****}p<6.87$  ⋅  $10^{-8}$; Welch’s t-test). Furthermore, averaging scores over WSIs generally resulted in lower Deletion scores than averaging across individual patches, highlighting the importance of aggregation method on performance evaluation.

**Table 1:** Mean individual patch AUC and WSI averaged AUC.

	Mean over WSIs		Mean over patches	
Method	Insertion *↑*	Deletion *↓*	Insertion *↑*	Deletion *↓*
Attention Rollout (without residual)	0.8170	0.5297	0.8449	0.6004
Attention Rollout (with residual)	0.8558	0.4412	0.8791	0.5318
Integrated Gradients	0.8559	0.6457	0.8766	0.7117
Random	0.7740	0.7685	0.8091	0.8034
RISE (binomial)	**0.9834**	0.2291	**0.9838**	0.2947
RISE (uniform)	0.9794	0.2626	0.9799	0.3378
ViT-Shapley	0.9740	**0.1866**	0.9751	**0.2511**

For *Insertion*, higher is better (*↑*); for *Deletion*, lower is better (*↓*).

### Statistical summary

Table 2 presents a statistical overview of the performance metrics, detailing mean AUC scores averaged per WSI and across all individual patches, respectively. In the table, p-values are shown for individual patch AUCs as sample populations and for per whole slide image averaged AUCs. However, it should be pointed out that each patient’s whole slide image provided a different number of patches. Thus, the samples are not i.i.d.

**Table 2:** Welch’s t-test’ p-values for Insertion and Deletion.

	Attn. Roll. (w/o res)	Attn. Roll. (with res)	integrated gradients	random	rise (binomial)	rise (uniform)	Shapley
	Insertion: Mean over per whole slide image averaged AUC						
Attn. Roll. (w/o res)	-	1	1	1	0	0	0
Attn. Roll. (with res)		-	1	0.42	0	0	0
Integrated Gradients			-	0.06	0	0	0
random				-	0	0	0
RISE (binomial)					-	1	0.22
RISE (uniform)						-	1
ViT-Shapley							-
	Deletion: Mean over per whole slide image averaged AUC						
Attn. Roll. (w/o res)	-	0.27	0.047	0	0	0	0
Attn. Roll. (with res)		-	0	0	0	0	0
Integrated Gradients			-	0.01	0	0	0
random				-	0	0	0
RISE (binomial)					-	1	1
RISE (uniform)						-	0.3
ViT-Shapley							-
	Insertion: Mean over all individual patch AUCs						
Attn. Roll. (w/o res)	-	0	0	0	0	0	0
Attn. Roll. (with res)		-	1	0	0	0	0
Integrated Gradients			-	0	0	0	0
random				-	0	0	0
RISE (binomial)					-	0	0
RISE (uniform)						-	0
ViT-Shapley							-
	Deletion: Mean over all individual patch AUCs						
Attn. Roll. (w/o res)	-	0	0	0	0	0	0
Attn. Roll. (with res)		-	0	0	0	0	0
Integrated Gradients			-	0	0	0	0
random				-	0	0	0
RISE (binomial)					-	0	0
RISE (uniform)						-	0
ViT-Shapley							-

Significant values are marked in red.

#### Computational complexity.

**Fig 5:**
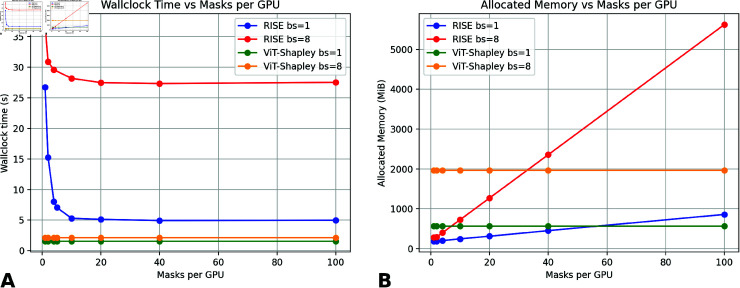
Comparative analysis of wallclock time and allocated GPU memory in RISE. (A) Wallclock time in relation to the number of masks per for-loop when Monte Carlo sampling in RISE. (B) Allocated Memory (GPU) in relation to the number of masks per for-loop when Monte Carlo sampling in RISE.

We benchmarked RISE and ViT-Shapley on a single NVIDIA GeForce RTX 2080 Ti with 20 CPU Cores (Intel(R) Xeon(R) Silver 4210 CPU @ 2.20GHz). Removal-based explainability methods and metrics typically rely on the classifier being robust to the distribution shift arising from masking of the input images. As this is usually not the case by default, applying removal-based explainability methods often comes with a preceding step of training a surrogate model matching the classifier under this perturbation. The surrogate model’s underlying backbone is a ViT-Small with a patch size of 16 imported from the timm library (vit_small_patch16_224). A comparison for compute and memory requirements of RISE and ViT-Shapley can be found in Fig 5. ViT-Shapley is by order of magnitudes faster with a runtime of 1.547 seconds for a single patch while RISE takes 4.978 seconds when fully parallelizing the sampling. With our hardware constraints, we found 10 masks per GPU and a batch size of 96 for RISE to be a good trade-off between *processing time of a single batch* vs. *throughput* which required 317 seconds. In our test set, the median number of tumor patches occuring in a WSI was 283 patches. Thus, processing such a WSI would take about 16 minutes. On the other hand, for ViT-Shapley, we could fit a batch size of 32 onto our GPU leading to a total processing time of about 14 seconds.

## Discussion

When assessing the effectiveness and usefulness of explainability methods, it is first important to set clear expectations: effective methods should highlight the factors that a classifier relies on to make its decisions, which may *match* human expectations or reveal *biases*, with both being equally important. Interpreting the results of explainability methods is therefore a complex task. Nevertheless, if a classifier demonstrates high quantitative performance in distinguishing between classes like *tumor* or *non-tumor*, it is reasonable to expect that it has identified significant features of those classes – and thus these features should be reflected by the explainability method and should be in line with the expectations of human experts for *obvious* cases. On the other hand, if a classifier tends to overfit to certain meaningless patterns in the training data, this should be a result of the explainability method just as well. For instance, our classifier was trained to distinguish between lymph node metastasis and normal tissue. It could be the case that the classifier learned from other features – not just the tumor cells but also from surrounding tissue or even irrelevant artifacts. Therefore, distinguishing between useless artifacts and important features is crucial to understand what the model relies on for its decisions. To thoroughly assess the effectiveness of the explainability methods in making this distinction, we evaluated them both qualitatively and quantitatively and put the results into clinical perspective.

### Qualitative and quantitative evaluation.

The observed artifacts in Attention Rollout, characterized by random dots of high perceived importance, negatively impact not only the quantitative outcomes but also the interpretability of resulting heatmaps. These artifacts render the attribution maps confusing, potentially obscuring meaningful insights. In contrast, Integrated Gradients presents a more visually coherent approach by highlighting individual tumor cells. However, it does not perform as well quantitatively, particularly in comparison to removal-based methods. This discrepancy suggests that, while Integrated Gradients may appear reasonable to the human eye, it may not fully capture the network’s reasoning process. Therefore, attribution metrics that bypass human biases are needed to accurately evaluate the quality of attribution maps. Nevertheless, relying solely on Insertion and Deletion metrics introduces its own form of inductive bias: Firstly, the concept of *removing information* as a means to assess importance is an artificial construct that may not align with the AI model’s operational principles. Removal-based methods incorporate this concept inherently in their design, in contrast to other methods that do not presuppose any specific mechanism of information assessment. Secondly, Insertion and Deletion ignore the relativity of attribution w.r.t. the ranked information. Thirdly, these metrics do not account for the correlation between patches or the network’s robustness, potentially overlooking the nuanced ways in which AI models derive their classifications. For instance, a network’s focus on a particular tumor cell might be identified as the primary explanation for a classification. However, the model’s ability to maintain accurate classification upon the removal of that cell – by shifting focus to another tumor cell – challenges the reliability of such metrics in isolation.

This complexity necessitates a balanced approach that combines metric-based evaluations with qualitative assessments of attribution maps or a better scalar measure of attribution. In this context, RISE and ViT-Shapley methods emerge as robust options, offering attribution maps that are both quantitatively and qualitatively trustworthy. While RISE demonstrates competitive outcomes, its utility is tempered by significant inference times, as it requires thousands of forward passes during inference (process of using the trained model to generate outputs or explanations). This is attributed to the Monte Carlo sampling process, which involves generating many random masks to estimate feature importance. While RISE could be feasible with parallelization in well-equipped environments, the slow inference times could limit its practicality in clinical settings that may lack the necessary GPU resources. Conversely, the ViT-Shapley method achieves an optimal balance between trustworthiness and efficiency. After an initial investment in training an explainer network (which takes approximately 3 days), the ViT-Shapley method enables efficient inference with a single forward pass (approximately 1.547 seconds for a single patch). This efficiency, combined with the method’s reliability, positions approximate Shapley values as a compelling solution for scaling explainability to Vision Transformers.

### Clinical implications.

Convolutional Neural networks (CNNs) have long dominated medical imaging, particularly histopathology, due to their easy-to-assess explainability, even though their performance is often surpassed by newer models like Vision Transformers. Clinicians have favored CNNs because their decision-making process can be more easily explained and validated. However, in our study, we evaluated several xAI methods applied to Vision Transformers, including Integrated Gradients, approximate Shapley values, and Attention Rollout, and found that ViT-Shapley provides highly interpretable and clinically relevant explanations for transformer models. This breakthrough in explainability enables the use of ViTs, which offer superior performance, in medical domains like histopathology. Advancing xAI in this field has the potential to revolutionize clinical workflows by making high-performance models both interpretable and clinically trusted, leading to more accurate diagnostics and better patient outcomes. By making transformer models interpretable with techniques like ViT-Shapley, we show their potential for clinical use, ultimately improving decision-making in histopathology.

### Future work.

Although this study focused on lymph node metastasis, future research could evaluate the performance of these explainability methods on a broader range of medical datasets to assess their generalizability. In particular, while our work studied xAI in the context of a classification task, there are other critical tasks, such as regression (e.g., mortality prediction), that may offer additional insights about how well these methods apply across different problem types. Additionally, developing more efficient methods with simpler training processes would be beneficial for gaining wider acceptance of AI models in healthcare, where practical usability and fast inference times are critical.

#### Materials and methods

##### Dataset description

The study utilized the CAMELYON16 dataset, consisting of 399 H&E stained WSIs of sections of sentinel lymph nodes and includes slides from 399 patients provided by two hospitals in the Netherlands in the first half of 2015: Radboud University Medical Center (RUMC) and University Medical Center Utrecht (UMCU). Of these patients, only two (cases 15 and 25) received neoadjuvant systemic therapy, and both had negative lymph nodes. Additional nodal status details are unavailable in the dataset. Expert pathologists provided non-exhaustive annotations for the slides. For training of the ViT-based classifier, we split the WSIs into a training set of 159 tumor-free and 111 tumor slides and a test set of 81 tumor-free and 48 tumor slides (with one WSI being corrupted). For WSI processing, our initial step involved identifying the regions containing tissue on each slide. We adopted a patch-based approach, where each WSI was split into smaller square sections referred to as *patches*. More specifically, we obtained 256×256 pixel patches from the identified tissue regions. This patch size has been selected in order to achieve a compromise between computational efficiency and the ability to capture sufficient histopathological details for subsequent analysis. For evaluation, we used 38 of the 48 tumor slides, omitting the top 10 slides with the most tumor patches to prevent the test set from being highly unbalanced. In total, the resulting test set consisted of 17,343 patches, providing a substantial and representative dataset for our analysis.

###### Preprocessing

We first locate tissue inside the WSI and then extract patches of size 256 × 256 that are found in the tissue area. We compute an image mask using the *colorization value* [20] defined as *c* ( *r* , *g* , *b* ) = | *r* − *m* | + | *g* − *m* | + | *b* − *m* | ,  where $m=\frac{r+g+b}{3}$. We then apply an adaptive threshold [22] to separate tissue from background. The colorization value of a pixel is lowest for all shades of grey and is high for all others. The WSIs were split on the basis of magnification 20 × .

####### Pretraining and model implementation details

In order to balance the training data, we perform undersampling on the majority class. We further employ data augmentation via uniform random modulation of brightness, contrast, saturation, hue and flipping. For pre-training of the backbone, we use ImageNet [23]. All models (classifier, surrogate and explainer) are trained on a batch-size of 64 using the Adam optimizer [24] with *β* = ( 0 . 9 , 0 . 999 )  and $\epsilon =10^{-8}$, and a learning rate of 2 . 5  ⋅  $10^{-5}$ for the classifier and surrogate and 2 . 5  ⋅  $10^{-4}$ for the explainer. At the beginning of the training, we warm-start the learning rate linearly followed by a cosine annealing without any restarts. The classifier and surrogate model are trained for 5 epochs while the explainer is trained for 10 epochs. With this setup on one NVIDIA GeForce RTX 2080 Ti, training the classifier takes about 22 hours and training the surrogate about 15 hours. We used two GPUs for training the explainer which took 2 days and 18 hours. However, there is a bottleneck stemming from loading the whole slide images in our hardware. Without that bottleneck, we expect training time to reduce significantly. The explainer training was limited to 10 epochs mainly due to computational budget constraints.

######## Attribution methods

In this study, we selected a set of explainability methods based on their strong performance in image classification tasks and their representation of diverse underlying approaches to model interpretability. We chose Integrated Gradients for its gradient-based attribution, Attention Rollout to leverage the inherent interpretability of attention mechanisms in Transformers, RISE for its robustness in perturbation-based attribution, and ViT-Shapley for its theoretical foundation in cooperative game theory, providing a comprehensive range of interpretability techniques from gradients, attention, perturbations, to game-theoretic methods. This subsection introduces aforementioned attribution methods from both an intuitive and theoretical perspective.

######### Integrated gradients.

The core concept of Integrated Gradients [15] is to assess how the network’s prediction changes as we move from a baseline input (such as a completely masked image) to the actual input of interest. This method calculates the path integral of the prediction function’s gradients along a straight-line path that connects the baseline to the input. This approach is grounded in the fundamental theorem of Calculus for line integrals, which can be expressed as follows:

Theorem 1. *Assume $f:{\mathbb{R}}^{d}\to \mathbb{R}$ is a differentiable function and $\gamma :[a,b]\to {\mathbb{R}}^{d}$ a smooth curve. Then*

$$\begin{array}{ccc} \int_{\left[a,b\right]}\frac{d}{dt}f(\gamma (t))dt=f(\gamma (b))-f(\gamma (a)). & & \end{array}$$ (1)

Given a baseline point $x^{\prime }$ and target point x, the straight line $\gamma :[0,1]\to {\mathbb{R}}^{d}$, $\gamma (t)=x^{\prime }+t\cdot (x-x^{\prime })$ is a smooth curve and Theorem 1 simplifies to


$$\begin{array}{llll} f(x)-f(x^{\prime }) & =\int_{\left[0,1\right]}\frac{d}{dt}f(\gamma (t))dt\; & & \;\\ & =\int_{\left[0,1\right]}{\nabla f|}_{\gamma (t)}^{⊤}\cdot \gamma^{\prime }(t)dt\; & & \;\\ & =\int_{\left[0,1\right]}{\nabla f|}_{\gamma (t)}^{⊤}\cdot (x-x^{\prime })dt\; & & \;\\ & =\overset{d}{\underset{i=1}{\sum }}(x_{i}-x_{i}^{\prime })\int_{\left[0,1\right]}\frac{df}{dx_{i}}(\gamma (t))dt.\; & & \; \end{array}$$

Every summand is now interpreted as the contribution of component *i* to transforming a baseline prediction $f(x^{\prime })$ into the actual prediction *f*(*x*), i.e.


$$\begin{array}{ccc} Int\text{\_}Grad(i):=(x_{i}-x_{i}^{\prime })\int_{\left[0,1\right]}\frac{df}{dx_{i}}(\gamma (t))dt. & & \end{array}$$ (2)

**Fig 6:**
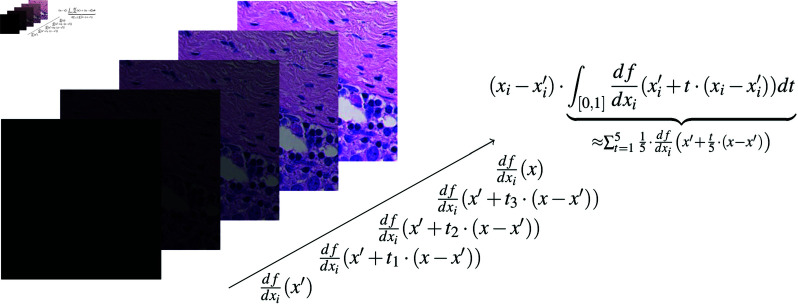
Integrated Gradients for *i*-th component. The line integral is approximated using a Riemann sum with *n* = 5 steps.

In case of image explanation, the baseline could be a completely masked image $x^{\prime }=0$ and each image patch would represent a component *i*. This is illustrated in Fig 6.

In practice, computing the integrals in exactly is infeasible. Thus, [15] propose to approximate the integral with a sample size that roughly satisfies the completeness axiom


$$\begin{array}{ccc} f(x)-f(x^{\prime })=\overset{d}{\underset{i=1}{\sum }}Int\text{\_}Grad(i). & & \end{array}$$ (3)

######## Attention rollout.

Attention Rollout tracks how information flows through different layers of a Vision Transformer. The method helps to explain how the Transformer makes decisions by tracing the contributions of individual tokens to the final prediction by investigating the attention maps of the different layers in the network [9].

Formally, during a forward pass, in each layer *l* = 1 , *…* , *n*, an attention map $A_{l}\in {\mathbb{R}}^{\left(d+1)\times (d+1\right)}$ is computed where *d* denotes the number of patches. Given the token’s value embeddings $v_{l-1}^{1},\ldots ,v_{l-1}^{d+1}$ from the previous layer, the next embeddings are retrieved in the following way:


$$\begin{array}{ccc} v_{l}^{i}:=A_{l}^{i}\cdot {\left[v_{l-1}^{1},\ldots ,v_{l-1}^{d+1}\right]}^{⊤}=\overset{d+1}{\underset{j=1}{\sum }}A_{l}^{ij}\cdot v_{l-1}^{j} & & \end{array}$$ (4)

**Fig 7:**
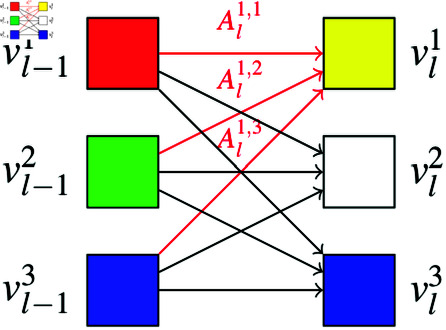
3 × 3 Attention Map interpreted as bi-partite Graph. The edge ($v_{l-1}^{j}$, $v_{l}^{i}$)’s weights corresponds to the corresponding value in the attention map. The yellow embedding results from retrieving half red, half yellow and zero blue.

This can be interpreted in the following way: The *i*-th row of $A_{l}$ represents how much information is retrieved from each of the previous token’s embedding. A visualization of this information flow as bi-partite graph is given in Fig 7.

**Fig 8:**
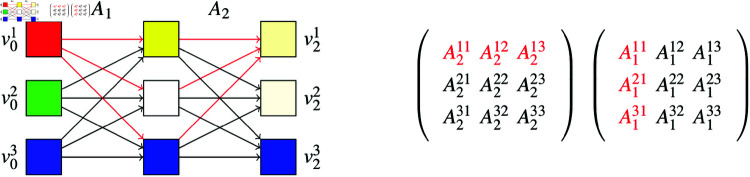
3 × 3 Attention Flow, 2 layers. There are 3 paths (marked in red) representing information flow from $v_{0}^{1}$ to $v_{2}^{1}$. The total amount of information flowing from source to target node is obtained through vector-vector multiplication of the red marked vectors.

Based on this observation, one could use the last layer’s attention weights pointing to the class token in order to explain which token contributes how much to the Vision Transformer’s prediction. However, it is unclear to which degree the embeddings of the last layer can be associated with the original input tokens. Instead, [14] propose to join these *l* bi-partite graphs respectively in their shared nodes as illustrated in Fig 8.

Formally, we obtain a graph *G* = ( *V* , *E* )  with

- nodes $V={⋃}_{l=0}^{n}\underset{=:V_{l}}{\underbrace{\left\{v_{l}^{1},\ldots ,v_{l}^{d+1}\right\}}}$
- edges $E={⋃}_{l=1}^{n}\left\{\left(v_{l-1}^{1},\ldots ,v_{l-1}^{d+1}\right)\right\}\times \left\{\left(v_{l}^{1},\ldots ,v_{l}^{d+1}\right)\right\}$
- edge weights $w:(v_{l-1}^{j},v_{l}^{i})\mapsto w_{l}^{ij}:=A_{l}^{ij}$.

Each path $p\in V_{0}\times \cdots \times V_{n}$ starting at node $v_{0}^{j}$ and ending in node $v_{l}^{i}$ represents information flow from token *j* to token *i* where the amount of information is measured by the product of all traversed edge weights


$$\begin{array}{ccc} flow((v_{0}^{i_{0}},\ldots ,v_{n}^{i_{n}})):=w_{0}^{i_{0},i_{1}}\cdot ...\cdot w_{n}^{i_{n-1},i_{n}}. & & \end{array}$$ (5)

Suppose the first token denotes the class token, i.e. *cls* : = 1. Attention Rollout quantifies the importance of token *i* as the sum of all flows from token *i* to the class token


$$\begin{array}{ccc} attention\text{\_}rollout(i):=\underset{p=(v_{0}^{i},\ldots ,v_{n}^{cls})}{\sum }flow(p)={\left[A_{n}^{cls}\cdot A_{n-1}\cdot \cdots \cdot A_{1}\right]}^{i}. & & \end{array}$$ (6)

While this tracks flow between attention modules, a Vision Transformer usually applies a skip connection after the attention module, i.e.


$$\begin{array}{ccc} v_{l}=A_{l}v_{l-1}+v_{l-1}=(A_{l}+I_{d+1})v_{l-1}. & & \end{array}$$ (7)

Thus, [14] extend by adding the identity matrix to each attention map $A_{l}$ and re-normalizing them row-wise. We denote this as Attention Rollout with residuals.

######## Randomized input sampling for explanation (RISE).

Attention Rollout provides explanations by tracking information flow through *attention layers*, making it Transformer-specific rather than *model-agnostic*. For more general use, black-box attribution methods, such as Randomized Input Sampling for Explanation (RISE) [16], offer a flexible alternative. These methods determine attribution by measuring fluctuations in the target class prediction *f* under image perturbation, making them applicable to any classifier regardless of its architecture.

RISE estimates the importance of a region in the input image via random masking of subparts of the image and measuring the change in prediction under this perturbation. A patch is considered important if the confidence of the model remains high even under masking of arbitrary other areas. More formally, a region or patch *i* is important if *f* is high in expectation over arbitrary masking of other regions, i.e.


$$\begin{array}{ccc} E[f(x⊙M)|M_{i}=1]↗1, & & \end{array}$$ (8)

where *x* is the input image and $M∼P_{M}$ a random variable mapping to ${\left\{0,1\right\}}^{d}$. We can re-write Equation in the following way:


$$\begin{array}{llll} E[f(x⊙M)|M_{i}=1] & =\underset{m\in {\left\{0,1\right\}}^{d}}{\sum }f(x⊙m)\cdot P(M=m|M_{i}=1)\; & & \;\\ & =\frac{1}{P(M_{i}=1)}\underset{m\in {\left\{0,1\right\}}^{d}}{\sum }f(x⊙m)\cdot P(M=m,M_{i}=1)\; & & \;\\ & =\frac{1}{P(M_{i}=1)}\underset{m\in {\left\{0,1\right\}}^{d}}{\sum }f(x⊙m)\cdot m_{i}\cdot P(M=m)\; & & \;\\ & =\frac{1}{P(M_{i}=1)}E[f(x⊙M)\cdot M_{i}]\; & & \;\\ & =\frac{1}{E[M_{i}]}E[f(x⊙M)\cdot M_{i}].\; & & \; \end{array}$$

**Fig 9:**
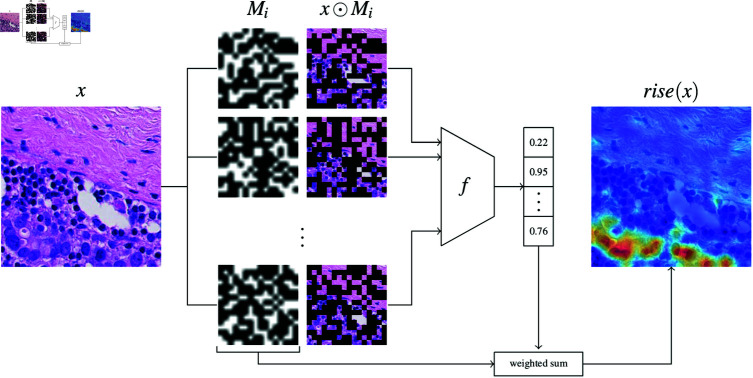
Randomized Input Sampling for Explanation (RISE). Monte Carle sampling with n masks $M_{1},\ldots ,M_{n}$.

Note that $P(M=m,M_{i}=1)=\{\begin{array}{cc} 0\; & \text{if }m_{i}=0\\ P(M=m)\; & \text{if }m_{i}=1 \end{array}\text{ since }\{M=m,m_{i}=1\}\subseteq \{M_{i}=1\}$. Thus, re-writing for every patch *i*, we obtain the following expectation in matrix notation


$$\begin{array}{ccc} rise(x)\approx \frac{1}{E[M]}E[f(x⊙M)\cdot M]. & & \end{array}$$ (9)

which is approximated using Monte Carlo sampling. The Monte Carlo sampling procedure is illustrated in Fig 9.

######## ViT-Shapley.

Shapley values provide a way to fairly distribute the total outcome of a cooperative effort by evaluating the contribution of each participant, i.e. a group of players working together to achieve a common goal. Shapley values quantify how much each player contributes to the group’s success. This concept, derived from game theory, is particularly useful for explaining complex models, as it can attribute the contribution of individual features to the overall prediction. ViT-Shapley approximates Shapley values for Vision Transformers, making the computation feasible for handling high-dimensional inputs. Therefore, a surrogate network is introduced, which approximates the model’s behavior when certain regions of the input image are masked or missing. Since classifiers are not typically trained on images with missing information, the surrogate network helps predict how the model would respond to such perturbed inputs. Building on this, an explainer network is then trained to assign Shapley values to different image patches. This network learns to determine which regions of the image contribute the most to the model’s prediction for each class, allowing for a clear explanation of how the Vision Transformer arrives at its decision.

######### Introduction of Shapley values.

In formal terms, Shapley values [25] have been introduced for *d*-player cooperative games to determine how much each player contributes to the group’s overall payout. A *d*-player cooperative game is defined via a set function $v:{\left\{0,1\right\}}^{d}\to \mathbb{R}$ satisfying *v* ( 0 ) = 0. Evaluating *v*(*m*) amounts to grouping a subset of players in a coalition ($m_{i}=1$  ⇔  player *i* is included), letting this coalition play out the game and obtaining their payout. The idea is now to compare for every coalition $m\in {\left\{0,1\right\}}^{d}$ its payout

a) with player *i*: $v(m+e_{i})$ vs.
b) without player *i*: *v*(*m*).

Player *i*’s Shapley value $\phi_{i}\in \mathbb{R}$ is then defined as


ϕi:=1d∑m∈{0,1}dmi=01d−11⊤ ⁡m (v(m+ei)−v(m)). (10)

Note that for fixed coalition size $k=1^{⊤}m$ we can draw d−1k different coalitions out of the player pool  { 1 , *…* , *d* } ∖ { *i* }  without player *i*. In other words, balances out term’s weights such that coalitions of size *k* have the same influence on the Shapley value as coalitions of size $k^{\prime }\neq k$. Unfortunately, computing is very expensive as it requires $2^{d}$ queries of *v*. [26,27] derived an alternative characterization as least squares solution of the following optimization problem


arg min ⁡ ϕ∈ℝdEpsh(m) [(v(m)−m⊤ ⁡ϕ)2]s.t.1⊤ ⁡ϕ=v(1), (11)

where


$$\begin{array}{ccc} p_{sh}(m)\propto \{\begin{array}{cc} (1^{⊤}m-1)!\cdot (d-1^{⊤}m-1)!\; & \text{if}\;0<1^{⊤}m<d\\ 0\; & \text{if}\;m=0\;\text{or}\;m=1 \end{array}. & & \end{array}$$ (12)

can now be solved using a projected stochastic gradient descent approach. To this end, [27] introduce additive efficient normalization


$$\begin{array}{ccc} \hat{\phi }=\left[\phi_{1}+\frac{v(1)-1^{⊤}\phi }{d},\ldots ,\phi_{d}+\frac{v(1)-1^{⊤}\phi }{d}\right] & & \end{array}$$ (13)

which projects back a candidate solution *ϕ* to the feasible space since


$$\begin{array}{llll} 1^{⊤}\hat{\phi } & =\overset{d}{\underset{i=1}{\sum }}\phi_{i}+\overset{d}{\underset{i=1}{\sum }}\frac{v(1)-1^{⊤}\phi }{d}\; & & \;\\ & =\overset{d}{\underset{i=1}{\sum }}\phi_{i}+v(1)-1^{⊤}\phi \; & & \;\\ & =v(1).\; & & \; \end{array}$$

Furthermore, [27] show that the resulting optimum does indeed solve . Bridging the gap to images, for any image *x* the goal is to explain a classifier $f:{\mathbb{R}}^{3\times \sqrt{d}\times \sqrt{d}}\to {\left[0,1\right]}^{C}$’s probability of a target class *y*. Thus, every patch of the image represents a player. The game $v_{xy}:{\left\{0,1\right\}}^{d}\to \mathbb{R}$ to be played amounts to evaluating the classifier on the image where the corresponding patches are masked out. Concretely,


$$\begin{array}{ccc} v_{xy}(m):=f_{y}(x⊙m)-f_{y}(0) & & \end{array}$$ (14)

where $f_{y}(0)$ denotes the baseline prediction, i.e. evaluating on the black image. Subtracting the baseline prediction is required to satisfy $v_{xy}(0)=0$.

######### Approximation of Shapley values.

Within the realm of Vision Transformer’s, [17] introduce an explainer network $\phi_{ViT}:{\mathbb{R}}^{3\times \sqrt{d}\times \sqrt{d}\times C}\to {\mathbb{R}}^{\sqrt{d}\times \sqrt{d}}$ which learns to predict the Shapley values regarding all classes *y* ∈ *C* for any image *x*. This is done by minimizing the loss


$$\begin{array}{ccc} \begin{array}{ccc} L(\theta )=E_{p(x,y)} & E_{p_{sh}(m)}\left[{\left(f_{y}(x⊙m)-f_{y}(0)-\phi_{ViT}(x,y;\theta )\right)}^{2}\right] & \\ & \text{s.t.}1^{⊤}\phi_{ViT}(x,y;\theta )=f_{y}(x)-f_{y}(0). \end{array} & & \end{array}$$ (15)

The explainer model $\phi_{ViT}$ applies additive normalization as outlined in after each forward pass, even during inference. So by default, the side constraint in is satisfied reducing the constrained problem to a plain minimization problem which can be tackled using using standard optimizers such as SGD, Adam [24], AdamW [28], etc.

Usually, classifiers are not trained on images with missing patch information. Thus, evaluating the classifier on masked images requires marginalizing over the missing patch information, i.e. computing


$$\begin{array}{ccc} f(x⊙m)=E[f(x)|x_{i_{1}}=X_{i_{1}},\ldots ,x_{i_{\left|1⊤m\right|}}=X_{i_{\left|1^{⊤}(1-m)\right|}}], & & \end{array}$$ (16)

where $i_{1},\ldots ,i_{\left|1^{⊤}(1-m)\right|}$ index the missing patches when masking with $m\in {\left\{0,1\right\}}^{d}$, i.e. $m_{i_{k}}=0$. To tackle this problem, [17] additionally train a surrogate model $g:{\mathbb{R}}^{3\times \sqrt{d}\times \sqrt{d}}\to {\left[0,1\right]}^{C}$ by minimizing


$$\begin{array}{ccc} L_{surrogate}(\eta )=E_{p(x)}E_{p(m)}\left[D_{KL}(f(x)∥g(x⊙m;\eta )\right], & & \end{array}$$ (17)

where


p(m)=1d1⊤ ⁡m⋅(d+1). (18)

Intuitively, sampling from can be thought of as uniformly sampling a cardinality *k* ∈ { 0 , *…* , *d* }  followed by drawing a mask *m* out of all dk possible masks with cardinality *k*. The surrogate model *g* then replaces *f* in .

#### Attribution metrics: insertion and deletion

Human inspection of qualitative examples is inherently biased by our intuition about *logically* appearing attribution maps. In that sense, neural networks may have a completely different way of perception and reasoning. This motivates the need for more mathematically grounded and objective metrics.

**Fig 10:**
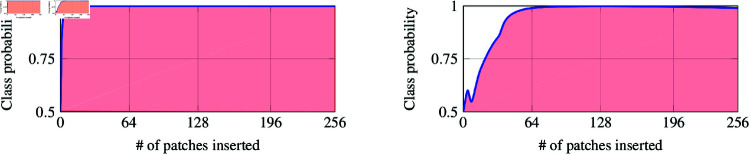
Classifier’s predicted class probability when adding patches step-by-step. (A) Important patches are inserted from the beginning leading to a high area under the curve. (B) Meaningless patches are inserted first. Thus, having a lower area under the curve.

When inspecting an image, humans usually only need a few select image patches to infer the corresponding object’s class. So, starting from a black image, if we were given the first few important patches step-by-step, our confidence in predicting the target class would quickly rise. Conversely, if given the wrong patches, i.e. with no useful information, we would remain rather unconfident in predicting the target class for a longer period of time. As a result, suppose we were to plot our confidence score *f*, i.e. predicted class probability, with regards to the number of inserted patches. The more meaningful the first few patches are, the quicker we would expect the curve to converge to a high probability. This is illustrated in Fig 10.

How quickly the curve converges to a high probability is then directly linked to the amount of area it covers. Mathematically, given the ranking *rank* : { 1 , *…* , *d* } → { 1 , *…d* }  resulting from argsorting the attribution map, **Insertion** measures the area under the curve of the plot


$$\begin{array}{ccc} \left\{\left(\#p,f(x⊙\overset{\#p}{\underset{i=1}{\sum }}e_{rank(i)})\right)|\#p=0,\ldots ,d\right\}. & & \end{array}$$ (19)

Higher is better. With the same intuition as above described in mind, we could also do this the other way around. That is, start with the whole image and step-by-step remove the patches according to the ranking in a descending order. If the first few patches were the most meaningful, then we would expect the class probability curve to quickly converge down to some low value. In other words, the covered area would be low this time. Mathematically, **Deletion** measures the area under the curve of the plot


$$\begin{array}{ccc} \left\{\left(\#p,f(x⊙\left[1-\overset{\#p}{\underset{i=1}{\sum }}e_{rank(i)}\right])\right)|\#p=0,\ldots ,d\right\}. & & \end{array}$$ (20)

Lower is better.
